# Regulation of CD38 on Multiple Myeloma and NK Cells by Monoclonal Antibodies

**DOI:** 10.7150/ijbs.68148

**Published:** 2022-02-21

**Authors:** Hao-Tian Wu, Xiang-Yu Zhao

**Affiliations:** 1 Peking University People's Hospital, Peking University Institute of Hematology, Beijing Key Laboratory of Hematopoietic Stem Cell Transplantation, National Clinical Research Center for Hematologic Disease, No. 11 South Street of Xizhimen, Xicheng District, Beijing 100044, China.; 2 Collaborative Innovation Center of Hematology, China.

**Keywords:** multiple myeloma, CD38, monoclonal antibody, NK cells, combination therapy

## Abstract

CD38 is highly expressed on multiple myeloma (MM) cells and plays a role in regulating tumor generation and development. CD38 monoclonal antibodies (mAbs) have been used as an effective therapy for MM treatment by various mechanisms, including complement-dependent cytotoxic effects, antibody-dependent cell-mediated cytotoxicity, antibody-dependent cellular phagocytosis, programmed cell death, enzymatic modulation, and immunomodulation. Although CD38 mAbs inhibit the proliferation and survival of MM cells, there are substantial side effects on antitumoral NK cells. The NK-mediated immune response needs to be further evaluated to minimize the adverse effects of NK cell loss. The killing effect of CD38 mAbs on CD38^high^ NK cells should be minimized and the potential combination of CD38^low/-^ NK cells and CD38 mAbs should be maximized to better benefit from their therapeutic efficacy against MM. CD38 mAb effects against MM can be maximized by combination therapies with immunomodulatory imide drugs (IMiDs), proteasome inhibitors (PIs), anti-programmed death 1 (PD-1)/programmed death ligand 1 (PD-L1) antibodies, or cellular therapies for the treatment of MM, especially in patients with relapsed or refractory MM (R/R MM) and drug-resistant MM.

## Introduction

Multiple Myeloma (MM) is hematological cancer with B cell lineage disorder, characterized by the expansion of malignant plasma cells in the bone marrow 1. Despite the use of high-dose chemotherapy in combination with autologous stem cell transplantation and other emerging new weapons, the prognosis of MM is disappointing. Novel therapeutic approaches have been tested in the last few years including new immunomodulatory drugs (IMiDs), proteasome inhibitors (PIs), and monoclonal antibodies (mAbs) 2, 3.

In 1980, during the pioneering analysis of the human lymphocyte surface, Reinherz and Schlossman discovered CD38, a 45-kDa peptide chain consisting of intracellular, transmembrane, and extracellular domains with two to four N-linked oligosaccharide chains of sialic acid residues 4. As a transmembrane glycoprotein with receptor-mediated adhesion function and ectoenzymatic activity, CD38 is ubiquitously expressed on MM cells, as well as some regulatory T cells (Tregs) and natural killer (NK) cells 5, 6. The characteristic high CD38 surface density in MM cells leads to the development of anti-CD38 mAbs 7. After decades of efforts, the aspiration to accurately target MM cells has begun to bear fruit in 2015, with Food and Drug Administration (FDA) approval of the CD38 mAb, Daratumumab 8. CD38 mAbs are transforming MM treatment in virtue of their distinct activity as a single agent or in combinations and the manageable toxicity 9.

At the meantime, a relatively high level of CD38 is expressed on NK cells, inducing antibody-dependent cellular cytotoxicity (ADCC) to kill tumor cells, raising a question of whether the CD38 mAbs have a positive or negative impact on NK cells and what strategy should be used to maximize immune effects against MM cells 9.

This review will focus on the tissue distribution and function of CD38, its structure, function, and treatment with CD38 mAbs. We will examine the role of NK cells in MM development and the effects of CD38 mAbs on NK cells. Finally, we will discuss the efficacy of CD38 mAbs in combination with other treatments to maximize their immune response.

## CD38 in MM

### CD38 distribution

CD38 is present on MM and NK cells, monocytes, and B and T cells, in descending order of CD38 expression level. It can serve as a membrane receptor, with its ligand CD31, to participant in endothelial adhesion. It also triggers signaling as a coreceptor with other membrane molecules including TCR and BCR complexes on lymphocytes and CD16 on NK cells 10. Besides these peripheral blood mononuclear cells (PBMCs), CD38 is expressed in nonhematopoietic cells as well, including prostatic epithelial cells, airway smooth muscle cells, and corneal suprabasal limbal epithelial cells 11-13. As a single chain glycoprotein with single transmembrane segment, the CD38 topological membrane orientation differs, in various tissues. In its most common type II orientation, CD38's catalytic domain faces the extracellular environment 14, 15, while in its type III orientation, the catalytic domain faces the cytoplasm 16, 17. The different orientations in different tissues have functional implications as enzymatic substrates can be consumed and products can be produced in extracellular or intracellular compartments 18.

Multiple myeloma patients have the highest level of CD38 expression (~10^5^) in plasma cells, followed by NK cells (~10^4^). In relapsed or refractory myeloma (R/R MM) patients, the CD38 expression levels are relatively high in NK cells, regulatory B cells (Bregs), Tregs and myeloid-derived suppressor cells (MDSCs) compared with CD8^+^ and CD4^+^ T cells, indicating that CD38 is involved in providing the immunosuppressive environment for tumor growth 19.

### CD38 as an ectoenzyme promotes MM proliferation

The ectoenzyme activities of CD38 vary considerably, three of which, nicotinamide adenine dinucleotide (NAD)^+^ hydrolase, adenosine diphosphate ribose cyclase (ADPRC), and cyclic adenosine diphosphate ribose (cADPR) hydrolase, were verified via anti-CD38 monoclonal antibody 20. The NAD^+^ hydrolase mediates the conversion of NAD^+^ to adenosine diphosphate ribose (ADPR). Although CD38 shares a common evolutionary ancestor with ADPRC, their enzymatic functions for the homeostasis of NAD^+^ and cADPR have evolved divergently. The ADPRC function is limited to generating cADPR from NAD^+^ (cyclase) while CD38 has multiple roles in the production and degradation of cADPR 21. CD38 also catalyzes the production of nicotinic acid adenine dinucleotide phosphate (NAADP) from nicotinamide adenine dinucleotide phosphate (NADP)^+^. Both ADPR and NAADP act as intracellular secondary messengers and play an essential role in Ca^2+^ influx by activating transient receptor potential melastatin-2 (TRPM2) in immunocytes to regulate many physiological processes 22. Moreover, the soluble extracellular domain of CD38 has been shown to mediate adenosine diphosphate (ADP) ribosylation of several proteins, including CD38 itself 23.

**Figure 1:**
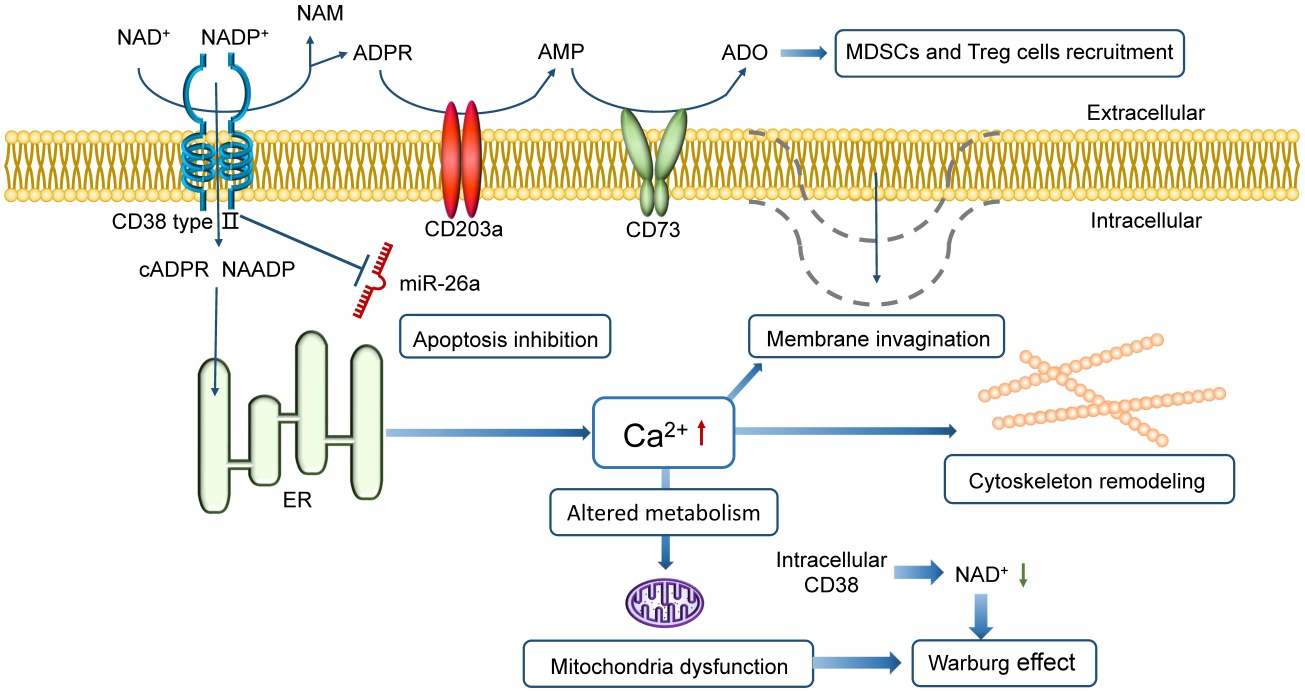
** Schematic representation of CD38 in promoting MM proliferation and preventing MM death.** CD38 directs the cyclization of extracellular NAD^+^ to intracellular cADPR (or ADPR) and triggers Ca^2+^ release to cytoplasm in MM cells. Intracellular CD38 contributes to a low level of NAD^+^ in cytoplasm. The high level of Ca^2+^ supports membrane invagination, cytoskeleton remodeling, and metabolic alteration. The high Ca^2+^ level in cytoplasm with mitochondria dysfunction and the low intracellular NAD^+^ level both leads to the Warburg effect. Besides, CD38 can protect MM cells from apoptosis caused by a miRNA, miR-26a. CD38 is also one of the three ectoenzymes in the CD38/CD203a/CD73 pathway that produces ADO, which not only regulates immune response in hypoxic conditions but also suppresses the antitumor immune response by recruiting MDSCs and Treg cells, inhibiting the activity of T effector cells and thus favoring tumor progression.

The NAD^+^-CD38-cADPR-Ca^2+^ axis, based on the ectoenzyme activity of CD38, regulates the tumor microenvironment by a series of reactions following Ca^2+^ flux. The high level of Ca^2+^ in cytoplasm supports cytoskeleton remodeling, plasma membrane invagination and Warburg effect (Figure 1) 24, 25. Targeting CD38 *in vivo* blocks mitochondrial transfer and improves animal survival in mice, demonstrating a consistently reduced tumor burden in bone marrows 26. It has been shown that the inhibition of CD38 expression could lead to tumor response, vital to achieving tumor control 27.

### CD38 prevents MM death

CD38 can protect tumor cells from all forms of cell death, including apoptosis, necrosis, and autophagy 28. It is an inhibitor of apoptosis in MM cells that blocks suppression of cell growth and apoptosis caused by miR-26a, a miRNA that induces apoptosis in MM 29. Besides, CD38 is one of the three ectoenzymes in the CD38/CD203a/CD73 pathway that produces adenosine (ADO), a nucleoside regulating immune response upon oxygen deficit environment. ADO also restrains the antitumor effect by MDSCs and Treg recruitment, inhibition of the activity of T effector cells and thus tumor progression. A higher expression level of CD38 in the tumor microenvironment is likely to confer a poorer prognosis 30, 31.

### CD38 functions in NK cells

**Figure 2:**
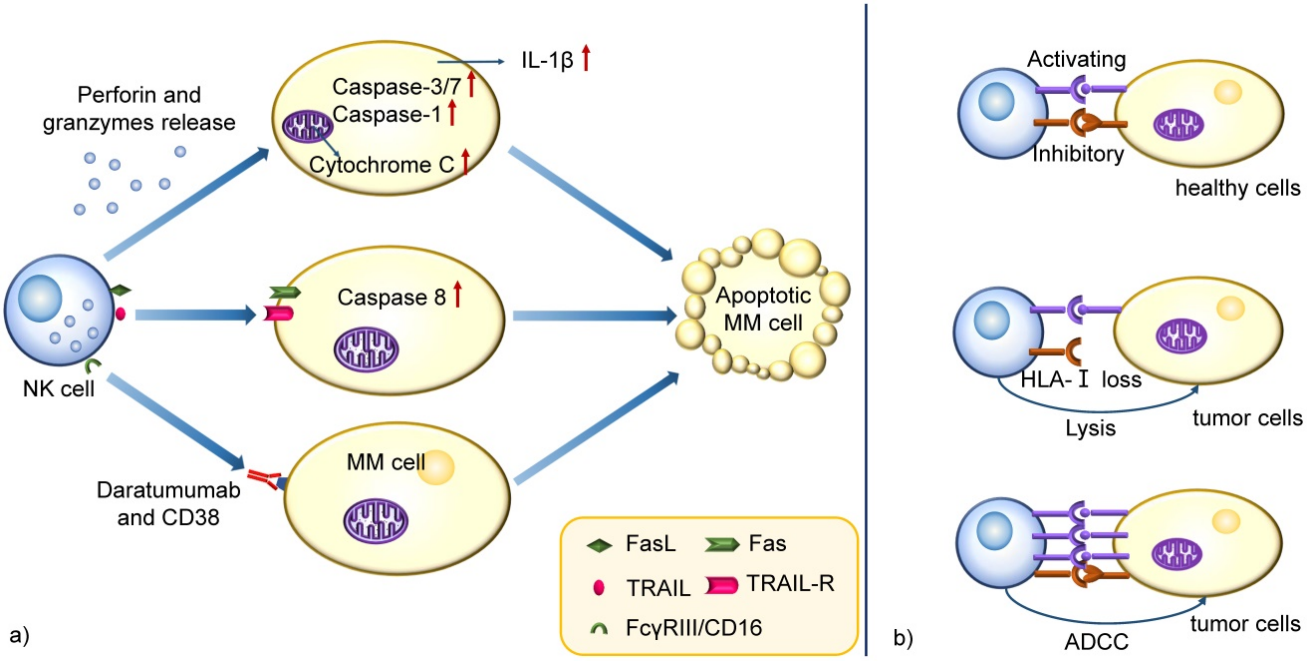
** Schematic mechanism representation of cytolytic functions in NK cells.** a) NK cells kill MM cells through perforin and granzymes release, death receptors activation and ADCC. The perforin and granzymes induce apoptotic cell death by different pathways, such as caspases-3/7 activation, cytochrome C release from mitochondria and IL-1β regulation by caspase-1. Another mechanism involves activating death receptors of TNFR superfamily expressed on target cells, which binds procaspase-8 after activation of their ligands on NK cells, allowing its activation and triggering apoptotic pathways. ADCC is another pathway to kill the target cells, especially when patients receive specific mAbs treatment. **b)** The cytotoxicity is dependent on the expression of activating and inhibitory receptors on NK cell membrane. They are engaged by specific ligands on target cells. The inhibitory ligands HLA-I are widely expressed on most healthy cells and the he inhibitory signal predominate the response in a healthy cell with a low quantity of activating ligands, blocking cell lysis. However, MM cells often downregulate HLA-I and allow effector NK cells to be activated by ITAMs in their cytoplasmic domains, resulting in the production of cytokines, granzyme B, and perforins and leading to tumor cell killing. Also, a strong capacity of ADCC is able to overcome these inhibitory signals.

NK cells are large granular effector lymphocytes of the innate immune system that can target tumor cells without prior sensitization. The primary mechanism of tumor killing via NK cells involves releasing cytotoxic molecules through a degranulation process. During the degranulation and release, perforin polymerizes and forms pores in the target cell membrane to facilitate granzymes delivery into target cells, inducing apoptosis through different pathways, including caspases-3/7 activation, cytochrome C release from mitochondria driven by Bak/Bax, and IL-1β regulation by caspase-1 32. Another mechanism involves activating death receptors of the tumor necrosis factor receptor (TNFR) superfamily which have high expression on target cells. Examples include CD95 (Fas) and TNF-related apoptosis-inducing ligand (TRAIL)-receptor (TRAIL-R) that are activated by their ligands, Fas ligand (FasL) and TRAIL, present on NK cell membranes or secreted by NK-derived exosomes. The oligomerized death receptors recruit Fas-associated death domain (FADD), which binds procaspase-8, allowing the tumor killing activation and spurring apoptotic pathways 33. Also, NK cells are armed with functional Fcγ receptor (FcγR) III/CD16. Thus ADCC is another pathway to kill target cells (Figure 2a) 34.

When an NK cell encounters a cell, it does not necessarily induce cell killing. Instead, the cytotoxicity relies on the expression of activating and inhibitory receptors on NK cell membrane, engaged by specific ligands expressed on target cells. Activating receptors include KIR2DS1, KIR3DS1, and natural cytotoxicity receptors (NCRs) and inhibitory receptors include KIR2DL1, KIR3DL1, and NKG2A 35.

It is known that almost all of the healthy cells in human body express the inhibitory ligands human leukocyte antigen class I (HLA-I). The inhibitory signal will predominate the response in a healthy cell with a low quantity of activating ligands, blocking cell lysis 36. However, tumor cells often downregulate HLA-I and allow effector NK cells to be activated by immunoreceptor tyrosine-based activation motifs (ITAMs) in intracellular domains, resulting in the release of cytokines, perforins and granzyme B and leading to tumor cell death. A strong capacity of ADCC is also able to overcome these inhibitory signals. (Figure 2b) 37, 38.

The resistance of MM cells to NK-mediated lysis is mainly associated with HLA-I-dependent mechanism. A high level of HLA-I on MM cell membranes may be involved in protecting these cells from NK-mediated attack and contribute to the immune evasion 39.

It is the loss of NK functional abilities rather than decreased numbers that results in monoclonal gammopathy of undetermined significance (MGUS) for MM progression 40. The malignant progression is accompanied with cytotoxicity decrease in NK cells and conversion to exhaustion due to changes in NK receptors, for example, the downregulation of NKG2D, NKp30, and DNAX accessory molecule-1 (DNAM-1) receptors. Additionally, a higher expression of programmed death 1 (PD-1) on NK membranes and the binding to its ligand on MM cells contribute to the impaired immune response and MM progression 41.

**Figure 3:**
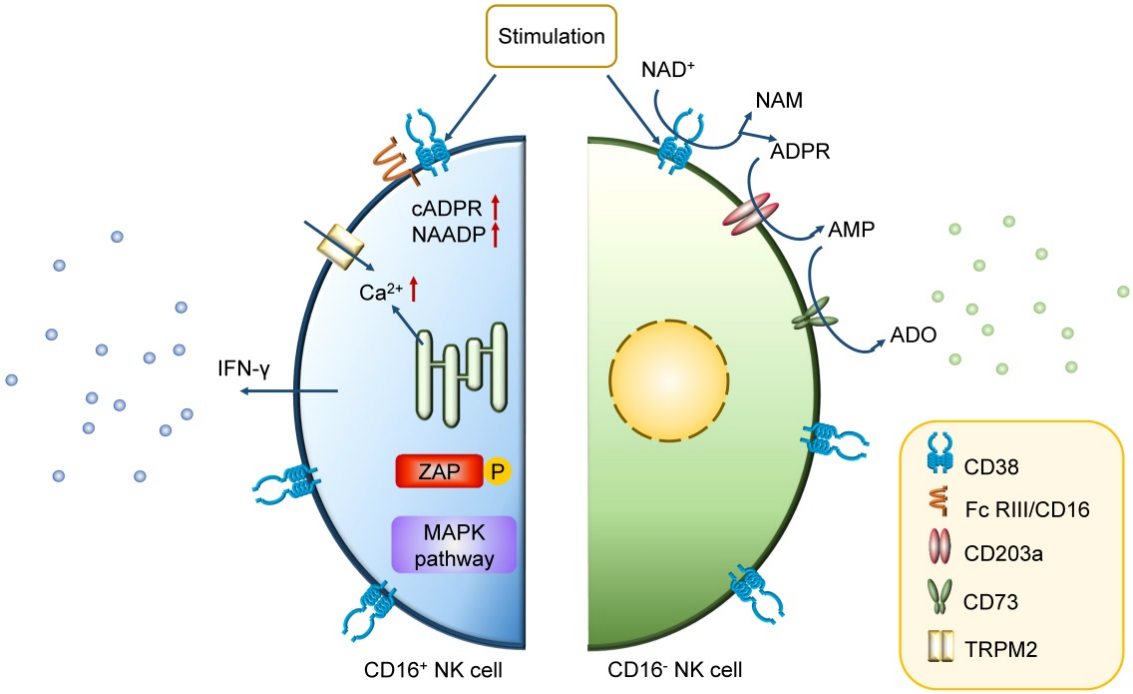
** Mechanism of action of CD38 in CD38^high^ NK cell.** In CD16^+^ NK cells, the association with CD16 is instrumental for the CD38-mediated intracellular signaling events. The high level of Ca^2+^ induces ZAP70 tyrosine phosphorylation, MAPK activation, and IFN-γ secretion. While in CD16- NK cells, the main function of CD38 is on the membrane. It mediates the production of adenosine, which induces the inhibition of CD4^+^ T cells, bringing the negative effect in immune response.

The early phases of hematopoietic differentiation and commitment towards the common lymphoid progenitor stage require CD38 expression together with other specific markers 42. In later stages of immuno-hematopoietic cells, some differences exist in the CD38 expression pattern. While some of mature cells start to decrease the expression of CD38, the majority of NK cells constitutively express CD38 43. It is physically or functionally clustered on these so-called conventional NK (cNK) cells with high level of CD38 as well as low-affinity receptor Fc RIII/CD16 44-46. The interaction between CD38 and some particular molecules is needed for initiation of specific transcription, secretion of cytokines, and activation of lymphocytic effector functions 45, 46. For conventional CD16^+^ NK cells, the association between CD38 and CD16 promotes the CD38-mediated activation of intracellular signaling pathways, resulting in the initiation of Ca^2+^ flux, zeta-chain-associated protein kinase 70 (ZAP70) tyrosine phosphorylation, mitogen-activated protein kinase (MAPK) activation inducing IFN-γ secretion and cytotoxicity crucial for the immune response 47. The interleukin-2 (IL-2)-dependent protein expression and CD38 association with CD16 on CD16^+^ NK cells are also central for the effector cytotoxic phenotype of lytic machinery 43. Furthermore, CD38 in CD16^-^ NK cells mediate the production of adenosine, inducing the inhibition of CD4^+^ T cells that can be reverted by blockade with CD38 antibodies (Figure 3) 48. It is believed that the knockdown of CD38 mediates a significant change in aerobic metabolism, including mitochondrial respiratory capacity enhancement, NAD^+^ boosting and Ca^2+^ transport. Besides, recent studies also suggest that it induces a long-term persistence of memory-like cells 49, 50.

Recently, an outstanding subset of NK cells (g^-^NK cells) have entered our vision. They account for 3~10% of NK cells in healthy samples and vary from the conventional NK cells by a lack of FcεRIγ expression 51. While encountering Cytomegalovirus (CMV) infections, the proportion of g^-^NK cells will increase to 25~30% 52. Further studies have indicated that g^-^NK cells are persistent memory-like cells (4~9 months). These g^-^NK cells have a minimal level of CD38 on surface 51. Although CD38 is vital in cNK cells, it is not a necessary property during immune response such as ADCC against MM. Because of the defect in expression of activation markers including CD38, NKp46 and NKp30, g-NK cells act as memory-like cells, with less IFN-γ secretion than activated cNK cells, but have stronger antitumor potential than cNK cells after CD16 crosslinking 53.

## CD38 mAb in MM

### Structure of CD38 mAbs

Daratumumab and Isatuximab are conventional CD38-specific mAbs, both derived from human CD38-immunized mice. Daratumumab was generated from gene-altered mice carrying genomic loci encoding human IgH and IgL, while Isatuximab was obtained from wild-type mice 54. Daratumumab has been approved for monotherapy use 55. The chimeric antibody was produced by genetic fusion of the VH and VL domains of the mouse monoclonal antibody to human IgG1 and kappa domains, respectively 56.

A constrained peptide approach showed that the epitope of Daratumumab was targeted to two β-strands containing amino acids 233~246 and 267~280 of CD38, and the specific amino acid residues were identified, in particular serine at position 274. The Daratumumab epitope on CD38 was speculated to offer a favorable structural arrangement for hexamer formation, allowing efficient C1q docking and initiation of the classical complement pathway 57.

### CD38 mAbs induce complement dependent cytotoxicity

As the most effective inducer of complement dependent cytotoxicity (CDC), Daratumumab is currently believed as one of the most important mechanisms of MM cell killing in the clinic, efficiently inducing high levels of CDC at a low concentration in bone marrow microenvironment 58, 59. Therefore, Daratumumab emerged as the preferred CD38 mAb in the clinical treatment of MM 59, 60.

The classical CDC pathway starts with a binding of CD38-CD38 mAb complex and the classic complement-activating protein C1q. It leads to the generation of anaphylatoxins (C3a, C4a, and C5a), chemo-attractants, and opsonins. Additionally, terminal complement pathway activation generates membrane attack complexes (MAC), which build pores in the MM cell membrane, resulting in direct cell lysis 61, 62. The Daratumumab-mediated CDC depends on CD38 expression level, and a high CD38 expression on MM cell membranes strongly improves CDC activity of Daratumumab 63.

### CD38 mAbs induce ADCC

In the presence of PBMCs enriched for many NK cells, Ab-dependent killing via FcR^+^ effector cell is also considered a vital mechanism for immune therapy 64, 65. The 51Cr-release assay evaluates the capacity of mAb-induced effector cell-dependent lysis of MM cells 66 and provides evidence that the lysis is dose-dependent and effective in the presence of bone marrow-derived mesenchymal stem/stromal cells (BMSCs) and in patient-derived PBMCs 59.

It has been reported that CD32c and CD16 are the key FcγRs on NK cells and CD64 is mainly present on macrophages, DC cells, neutrophils, and eosinophils 67. MM cell death occurs via NK cell degranulation and release of perforins and granzymes and via activation of receptors inducing MM cell apoptosis 68, 69. The secretion of pro-inflammatory cytokines, such as interferon-γ (IFN-γ), TNFα, and granulocyte-macrophage colony-stimulating factor (GM-CSF), is triggered by CD16 engagement, contributing to the recruitment of adaptive immune cells 70. However, the ADCC lysis is reduced following Daratumumab treatment, probably due to decreased NK cell numbers. In most patients, PBMCs isolated during CD38 mAb treatment can still induce some level of ADCC, indicating that the remaining group of NK cells maintain cytotoxic functionality 71. ADCC is widely observed in CD38 mAb treatment and is the main mechanism of Isatuximab-induced tumor cell death in several lines of experiments. Its efficiency depends on CD38 expression 63, 72.

### CD38 mAbs induce macrophage-mediated phagocytosis

Human macrophage-mediated antibody-dependent cellular phagocytosis (ADCP) is another efficient killing mechanism of Daratumumab by rapid and sequential engulfing of multiple MM cells 54. The percentage of macrophages and the number of remaining MM cells are the main indicators for estimating the ADCP level of MM cells 54. Although ADCP-induced phagocytosis is related to CD38 level on targeted cells, differences in CD38 expression levels of different target cell lines do not appear to be a convincible explanation for the differences in phagocytosis-mediated elimination 73.

Phagocytosis is suppressed via CD47/SIRPα activation. CD47 on MM cells can bind to SIRPα on macrophages, resulting in an inhibition of phagocytosis 74. Recently, the neutralization of CD47 by its antibody has been shown to enhance ADCP of MM cells mediated by CD38 mAb 75-77. Also, low-dose cyclophosphamide is used to improve Daratumumab-mediated ADCP against tumor cells, probably mediated by increased expression of FcγR on macrophages and reduced CD47 levels on tumor cells 78. Moreover, it is also partly dependent on the ratio of monocytes to MM cells 63.

### CD38 mAbs induce programmed cell death

Programmed cell death (PCD) mediated by Daratumumab has been observed in MM cells 79. In CD38^+^ MM cell lines, PCD is induced by activating FcγRI-expressing cells cross-linking Daratumumab 79. During cell death, phosphatidylserine translocation is a reversible process and initiation marker, whereas damage of the mitochondrial membrane potential and cell membrane integrity are irreversible 79. Another anti-CD38 mAb, Isatuximab, has also shown significant antitumor activity by a more potent action of direct cell killing different from crosslinking, mediated by activation of caspases-7 and 8, lysosome permeabilization, upregulation of reactive oxygen species (ROS), as well as ADCC, CDC, and ADCP mentioned above 80, 81.

### CD38 mAbs modulate enzymatic functions

Daratumumab mainly inhibits the cyclase activity and hydrolyzes cADPR *in vitro*, increasing NAD and ADPR levels, decreasing Ca^2+^ mobilization, and reducing signaling in MM cells, all of which induce cell death 57. However, CD38 on tumor cells displays more NAD^+^ hydrolase than ADPRC activity. Thus, besides Daratumumab, a better CD38 mAb is required to inhibit the NAD^+^ hydrolase activity of CD38^+^ MM cells 82.

Another CD38 mAb, Isatuximab, almost completely inhibits the enzymatic activity of recombinant CD38 (5 nmol/L) at concentrations of 20~200 nmol/L, including its hydrolase activity 83. CD38 is also suggested as the dominant enzyme for ADO generation in MM cells, alleviating an immunosuppressive microenvironment in MM patients. However, the certain evidence whether the inhibition of ADO-mediated signaling pathway has a direct impact on anti-MM activities of CD38 mAbs is still confusing because of the technical challenges of direct measurement of ADO level in patients 84, 85.

### CD38 mAbs induce immunomodulation

**Figure 4:**
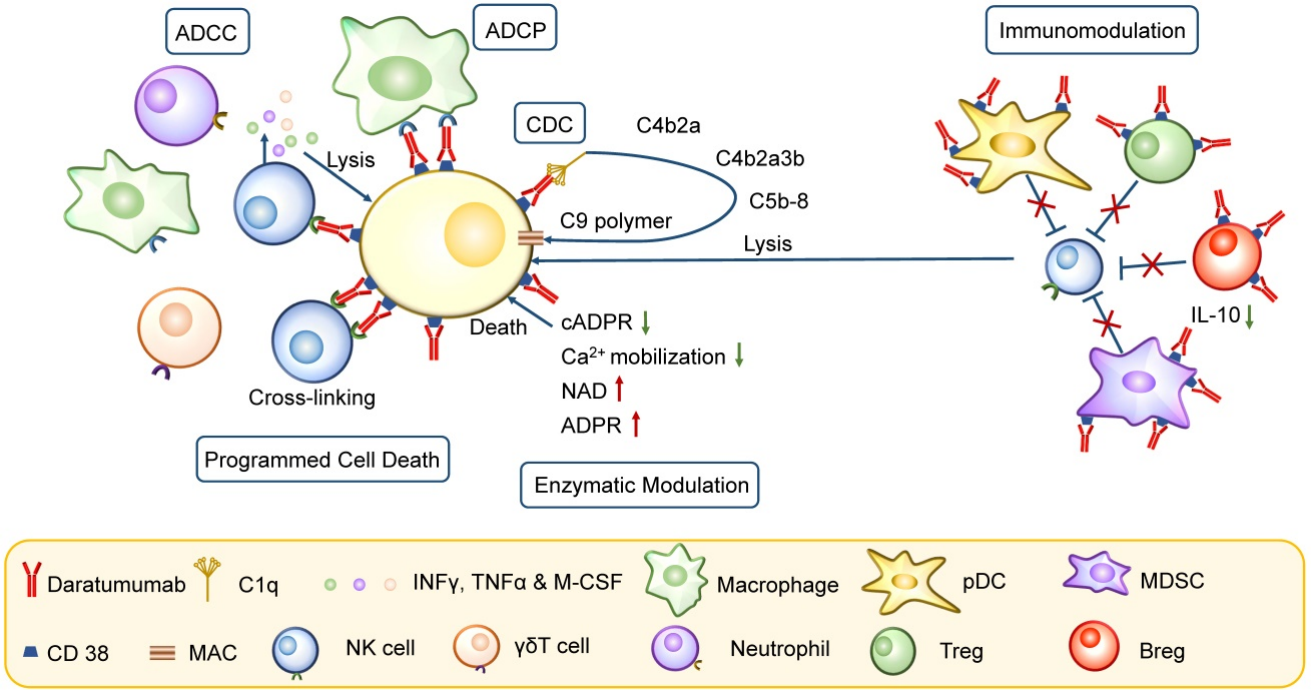
** Mechanism of action of CD38 mAbs.** CD38 mAb Daratumumab has pleiotropic mechanisms of action, including Fc-dependent immune-effector mechanisms, programmed cell death, enzymatic modulation and immunomodulation. The Fc-dependent immune-effector mechanisms consist of CDC, ADCC and ADCP. CDC is initiated by binding of C1q, leading to the activation of downstream complement proteins and resulting in assembly of the membrane attack complex (MAC); ADCC is achieved through the activation of FcRs on NK cells and other cells including neutrophils, macrophages and γδT cells; ADCP is mediated by macrophages and other immune cells, following interaction of the Fc frament of Daratumumab with FcRs on effector cells. Besides, PCD is activated by activating FcγRI-expressing cells cross-linking Daratumumab. In enzyme modulation, the reduction in cADPR and Ca^2+^ mobilization and the increase of NAD induce cell death. The immune dysfunction in MM is mediated by pDC, Tregs, MDSCs and Bregs. CD38 mAbs cause a depletion of these cells, which is crucial for effective immune response.

CD38 is widely expressed on immune cells and tumor cells. The CD38^+^ plasmacytoid dendritic cells (pDCs) confer a growth and survival advantage to MM cells by decreasing the pDC antitumor function 86. Daratumumab induces progressive depletion of pDCs and prevents upregulated programmed death ligand 1 (PD-L1) expression on APCs to improve the disease response 87. Besides pDCs, immune dysfunction in MM is also mediated by MDSCs, Tregs, and Bregs. The Tregs can be divided into 2 subpopulations, CD38^+^ Tregs that make up approximately 90% of the total Treg population and are more potent in suppressing T cell proliferation, and CD38^-^ Tregs. Daratumumab eliminates the subset of CD38^+^ Tregs, while Tregs without CD38 expression are not affected 5. MDSCs are also effectively killed by Daratumumab, as observed in CDC and ADCC assays 5. Bregs also contribute to an immunosuppressive microenvironment by producing and releasing IL-10 and abrogating NK cell-mediated ADCC 88. Since CD38 is present on these immunosuppressive cells, Daratumumab significantly induces immunomodulation during response against MM 88. Daratumumab causes depletion of CD38^+^ Tregs, Bregs, and MDSCs and an increase in clonality and effector functions of T helper and T cytotoxic cells 89. Isatuximab causes preferential depletion of Tregs by causing apoptosis, inhibiting cell proliferation, and relieving conventional T cells from suppression, thereby inducing immunomodulation in MM patients (Figure 4) 80, 81.

### Effects of CD38 mAbs on NK cells

Although the CD38 mAbs have positive effects on MM cells, they can also interfere with immune cell populations, and their effect on NK cells is unclear and controversial 90.

**Figure 5:**
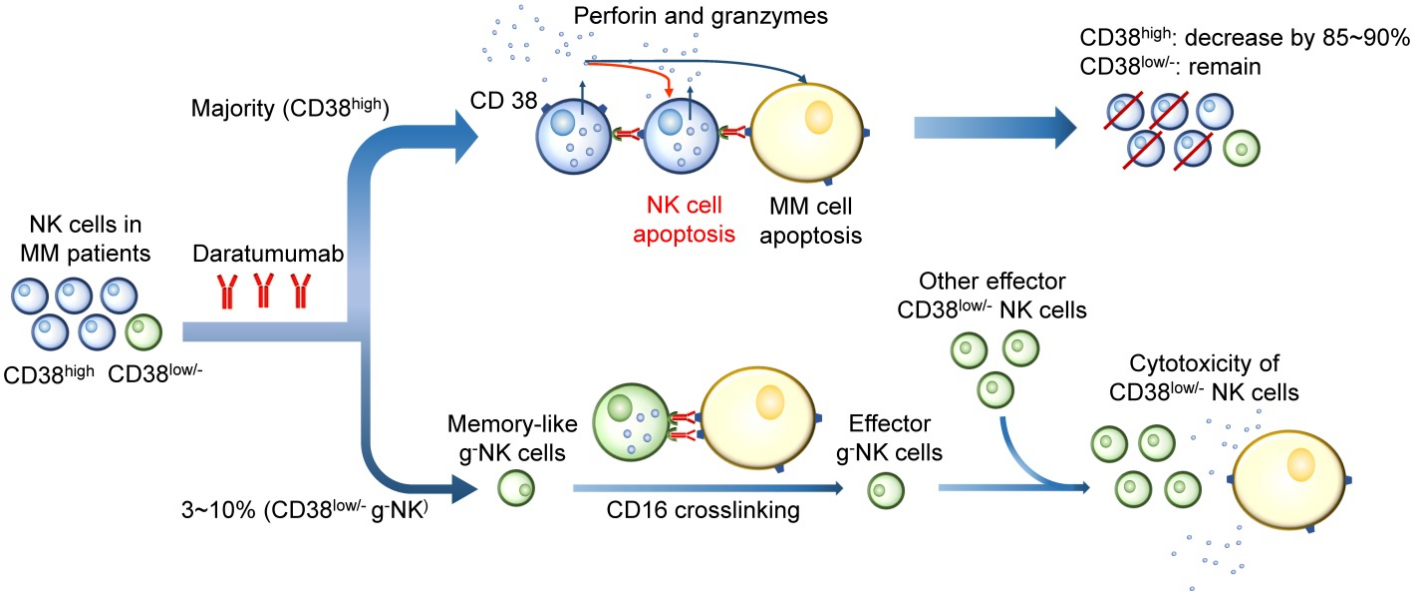
** Effects of CD38 mAbs on CD38^high^ and CD38^low/-^ NK cells.** CD38 mAbs are believed to cause an impaired immune response mediated by NK cells. Since CD38 is highly expressed on the majority of NK cells, Daratumumab induces NK cell apoptosis by fratricide or NK-mediated cytotoxicity by ADCC. A large quantity (85~90%) of CD38^high^ NK cells are killed. However, the residual CD38^low/-^ NK population displays a high proliferative potential and functional activity. The memory-like g^-^NK cells are activated after CD16 crosslinking. The effector CD38^low/-^ NK cells show a high proliferative potential and functional activity in the presence of CD38 mAb by inducing ADCC in the stressed tumor microenvironmental condition.

CD38 mAbs are believed to cause an impaired immune response, especially the response mediated by NK cells. Depletion of CD38^high^ NK cells has been observed in patients treated with Daratumumab 91. Studies have shown that Daratumumab triggers a dose-dependent NK cell apoptosis, resulting in a significant reduction in primary NK cells. A strong increase in NK cell degranulation has been observed without target MM cells, indicating that Daratumumab may induce NK cell apoptosis by NK cell fratricide or NK-mediated cytotoxicity by ADCC 5, 92. The ^51^Cr release assay and flow cytometry-based cytotoxicity assay indicated the existence of NK cells self-lyse, and Daratumumab integrity is necessary for the cytotoxicity 91. However, other observations suggest that the residual CD38^low/-^ NK population displays a high proliferative potential and functional activity. Therefore, CD38^low/-^ NK cells play a significant role in the presence of CD38 mAb by inducing ADCC in the stressed tumor microenvironmental condition 93. One of the uncertainties to predict the outcome of CD38 mAbs treatment is the g^-^NK cell. This subset of CD38^low/-^ NK cells markedly enhances ADCC against MM cells during the CD38 mAb treatment and have longer persistence than CD38^high^ cNK cells 94. These two observations are difficult to reconcile, since both processes occur in a single MM patient. Whether their heterogeneity and different response levels to CD38 mAb in patients contribute to different outcomes following Daratumumab therapy requires more evidence (Figure 5) 55.

As discussed above, NK cells play a significant role in antitumor response in MM patients. There are consequently several combined therapies based on NK cells, trying to increase antitumor efficacy via variant ways, including regulating NK cell ligands on MM cells, increasing the number and activity of effector NK cells and better utilizing the antitumor effect of CD38^low/-^ NK cells.

## Combined therapy based on NK cells

### CD38 mAb and immunomodulatory imide drugs

The combined therapy of CD38 mAbs with immunomodulatory imide drugs shows excellent performance in relapsed or refractory MM patients. The clinical use of IMiDs in MM patients has significantly improved their long-term survival and life quality. Thalidomide is widely used in newly diagnosed, relapsed, or refractory MM, especially as maintenance therapy after autologous stem cell transplantation (ASCT). Lenalidomide is a second-generation IMiD with fewer side effects in older patients than the first generation of IMiDs. Pomalidomide is a third-generation IMiD that is 10 times more potent than Lenalidomide and has shown impressive results in relapsed or refractory patients after Lenalidomide and Bortezomib therapy 95.

In the combined therapy, IMiDs increase the direct toxic effects of CD38 mAbs, including lysosomal-mediated apoptotic pathways, and increase CD38 expression on Treg cells, resulting in enhanced elimination of Treg cells 96, 97. Also, preclinical data show that IMiDs synergize with Daratumumab in patients with IMiD-resistant tumors, indicating that their immune system still responds to the immunomodulatory effects of these agents 98.

During IMiD treatment, the transcription factors Ikaros (IKZF1) and Aiolos (IKZF3) are degraded. A recent study has found that CD38 mRNA expression on MM cells is increased on Ikaros and Aiolos degradation after Lenalidomide treatment. Thus, an alternate cell-intrinsic hypothesis might explain the immunoenhancing effect of CD38 mAbs combined with IMiDs. It is inferred that the transcriptional activation of interferon-stimulated genes (ISGs), including CD38, results from signal transduction after IFN-α/β binding to the type 1 interferon (IFN) receptor, activation of janus kinase 1 (JAK1), signal transducers and activators of transcription 1/2 (STAT1/2) and interferon regulatory factor 9 (IRF9), assembly of the IFN-stimulated gene factor 3 (ISGF3) complexes that translocate to the nucleus and bind to IFN-stimulated response elements 99. Despite this, the CD38 expression on activated NK cells is not altered by Lenalidomide treatment despite a similar reduction in Ikaros, indicating that this process is specific to MM cells compared to immune cells 100.

**Figure 6:**
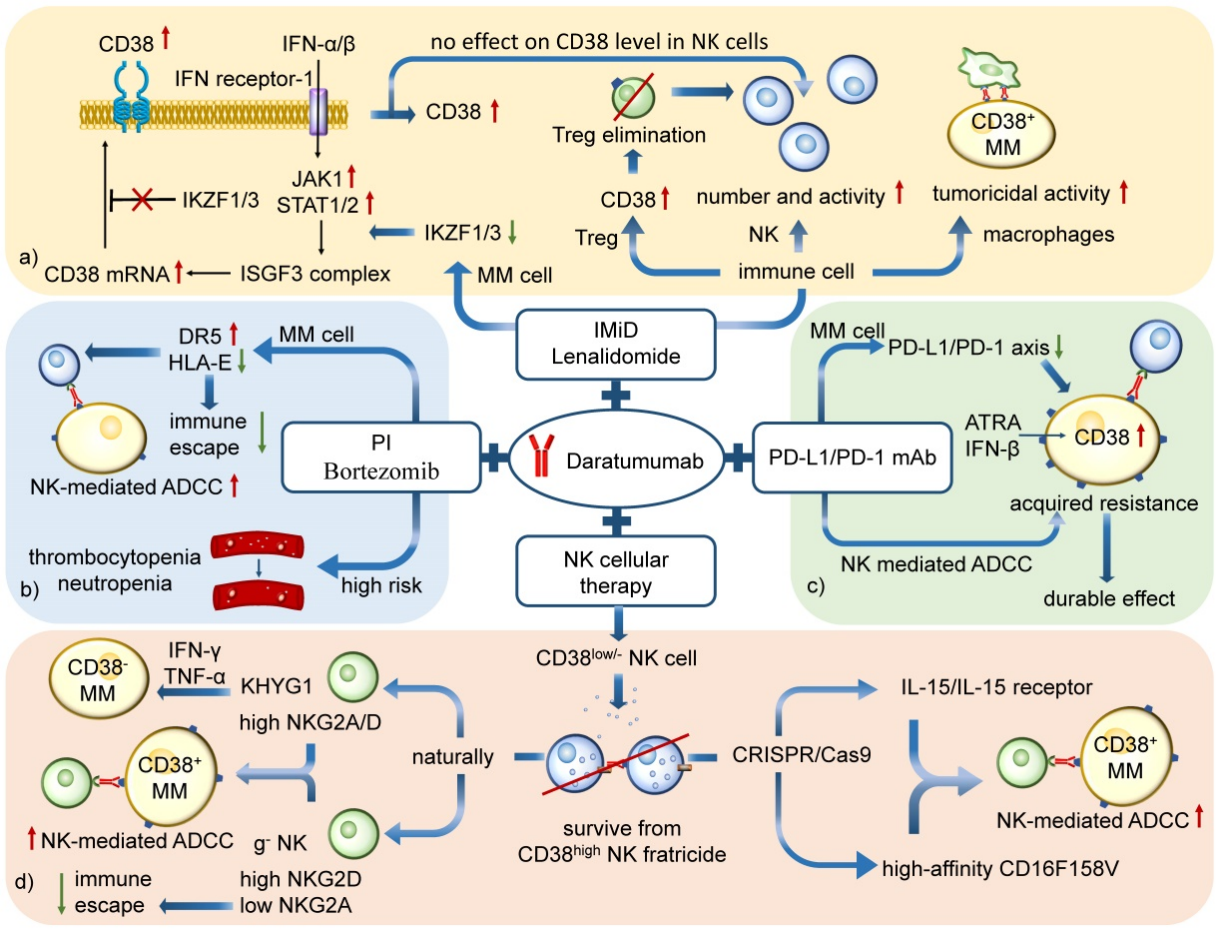
** Combined therapy against MM based on NK cells. a)** CD38 mAbs Daratumumab with IMiDs Lenalidomide: CD38 expression in MM cells is inhibited by transcription factors Ikaros (IKZF1) and Aiolos (IKZF3). IMiD reduces the IKZF1/3 and increases the CD38, which is benefit for Daratumumab treatment. The combined therapy markedly enhances NK cell-mediated ADCC by increasing the number and activity of NK cells and eliminating Tregs. It can also promote macrophage-mediated ADCP. **b)** Daratumumab with PIs Bortezomib: An increasing level of DR5 and decreasing level of HLA-E enhance NK-mediated ADCC and reduce immune escape. However, the combination is associated with infusion-related reactions and accounts for risks of thrombocytopenia and neutropenia. **c)** Daratumumab with PD-1/PD-L1 mAb: The downregulation of PD-L1/PD-1 axis in MM cells and the activation of ADCC in NK cells show potential on durable control in drug-resistant MM. **d)** Daratumumab with NK cellular therapy: The combination aims to enhance the tumoricidal effect of CD38^low/-^ NK cells. KHYG1 and g^-^ NK cells are CD38low/- NK cells which prove to be effective to kill MM cells by ADCC. G^-^NK cells are also able to reduce the immune escape because of its low level of NKG2A expression. Besides, the CD38^low/-^ NK cells engineered by CRISPR/Cas9 system is another method to maintain NK cell function during Daratumumab therapy.

Although CD38^high^ NK cell population is reduced by CD38 mAb, it brings few destructive effects to the efficacy and safety during combined therapy, because IMiDs, such as Lenalidomide, markedly enhance NK cell-mediated ADCC by increasing the number and activity of activated CD16^+^ NK cells 71, 98, 101. In addition, IMiDs also enhance CD38 mAb-mediated ADCP by promoting the tumoricidal activity of macrophages 102. The IMiD-mediated recovery of CD16^+^ NK cells makes the combined therapy possibly a good choice to conquer the negative affect of reduction of CD38^high^ NK cells in CD38 mAbs (Figure 6a) treatment 103.

### CD38 mAb and proteasome inhibitors

The combination of Daratumumab with proteasome inhibitors is beneficial in patients, including those over 65 years old and those with prior PI Bortezomib exposure. Bortezomib sensitizes MM cells to NK cells via upregulation of death receptor 5 (DR5) and selective reduction of HLA-E on MM cells, reducing the HLA-E-mediated immune escape in MM patients 104. It is believed that PIs enhance the therapeutic efficacy of Daratumumab by sensitizing tumor cells for NK-mediated ADCC and improving MM cell lysis. The rate of overall response, minimal residual disease-free survival and progression-free survival rate are significantly higher in relapsed and refractory MM patients receiving combined therapy than those treated with PIs only 105, 106. While the clinical evidence strongly suggests that the outcome of combined therapy is better than single drug therapy, there are few researches paying attention to the clinical efficacy in patients with different proportion of CD38^high^ and CD38^low/-^ NK cells.

However, it is of note that the combined therapy is associated with infusion-related reactions and accounts for higher rates of thrombocytopenia and neutropenia than treatment with bortezomib or dexamethasone alone, which may cause harmful effects to patients (Figure 6b) 107.

### CD38 mAb and PD-L1/PD-1 mAb

PD-1 is a 288 aa type I transmembrane protein and belongs to the CD28 receptor family. PD-L1, also known as B7-H1 and CD274, is a 40kDa type I transmembrane glycoprotein. The engagement of the PD-1 receptor with PD-L1 activates PD-1 downstream from Src homology region 2 domain-containing protein tyrosine phosphatase (SHP-2) and dephosphorylates ZAP70, leading to the inhibition of T cell survival, proliferation and cytokine production, inducing T-cell exhaustion; it also promotes Tregs development and decreases NK cell cytotoxicity, granule exocytosis, and IFN-γ secretion through the pathway by protein kinase C (PKC), phosphoinositide 3-kinase (PI3K) , extracellular-signal-regulated kinase (ERK), and protein kinase B (AKT) activation 108, 109. PD-L1 expression plays a dominant role in MM cells in immune inhibition and immune escape 110-112.

The anti-PD-1/PD-L1 antibody inhibits the negative impact of the PD-L1/PD-1 axis on treatment. However, previous data demonstrated that the tumor growth suppression and metastases by anti-PD-L1 antibody treatment lacked a complete durable response 110, 113, suggesting the existence of resistance mechanisms. Studies have demonstrated that the resistance indeed developed progressively during 5~7 weeks of PD-L1/PD-1 antibody treatment. Among the top 200 differentially-expressed genes, CD38 was identified as the only prominently upregulated gene/protein 114. The level of CD38 mRNA and protein was significantly increased on anti-PD-L1 resistant tumor cells. The hypothesis for PD-1/PD-L1 blockade focuses on the infiltration of activated T cells and inflammatory changes that lead to all-trans-Retinoic acid (ATRA)- and IFN-β-mediated CD38 upregulation, which mediates immunosuppression via adenosine production and its effect on CD8^+^ cytotoxic T cells 114.

Interestingly, it has also been shown that increased expression of the PD-1/PD-L1 axis suppresses the activity of NK cells, the CD38 mAbs-mediated ADCC 115. CD38 mAb Isatuximab, but not Daratumumab, blocks CD38 enzymatic activity of adenosine production 72. Thus, the combined therapy of two kinds of antibodies is beneficial to MM patients, especially those who received anti-PD-1/PD-L1 antibodies and developed acquired resistance 114, 116. Also, pre-treatment with clinically relevant agents such as ATRA and Panobinostat could be used to enhance CD38 expression. Preclinical studies to evaluate this treatment are in Phase Ⅰ trials 63, 117, 118. Recently researches have confirmed a better Daratumumab-mediated cytotoxicity when a PD-L1/PD-1 mAb is added to single Daratumumab treatment, showing another advantage for the combined therapy 119. The future is promising for this therapy (Figure 6c).

### CD38 mAb and NK cellular therapy

CD38 plays a significant role in NK cell function by facilitating ADP-ribose pyrophosphate (ADRP) production and mobilization of intracellular Ca^2+^ necessary for cytolytic degranulation. However, the treatment with Daratumumab leads to a rapid elimination (85~90%) of CD38^+^ NK cells, lasting up to 6 months after the treatment 94, 120. Since CD38^low^ NK cells display cytotoxic superiority in NK-mediated antitumor response, their efficacy should be maximized 91.

It is reported that different NK cell subsets differ in the level of Killer-cell immunoglobulin-like receptors (KIR) surface expression that has a negative impact on Daratumumab-mediated ADCC 93. Compared with primary expanded NK and NK-92 cells with high expression of CD38, KHYG1 cells have significantly lower CD38 surface expression. It is believed that low CD38 expression is enough to initiate the intracellular signaling pathways since other receptors, such as NKG2D on KHYG1 cells, can compensate for the lack of CD38-mediated effector functions 63. A study on three different NK cell lines suggests that compared to primary expanded cells with high level of KIR2DL1, KIR2DL2/3, and KIR3DL1, the NK-92 and KHYG1 NK cell subsets have low cell surface expression of the inhibitory receptors but a high proportion of NKG2A, important for NK cell education. Besides, KHYG1 expresses a natural variant of low-affinity CD16, which is called CD16F158V. It is a high-affinity receptor which markedly enhances the tumoricidal immune response in the CD38low/- NK cells 121. It is also shown that KHYG1 NK cells can be used in combination with CD38 mAb to specifically target and kill CD38^high^ and even CD38^low^ MM cells *in vitro*, possibly by releasing IFN-γ and TNF-α 122. Therefore, KHYG1 NK cells could be considered as an NK cell pool with low CD38 expression that can survive from Daratumumab-mediated NK cell decrease while still capable of mounting a cytotoxic response against MM target cells. Furthermore, given that the patients with high pre-treatment CD38 levels on MM cells have a better response to Daratumumab monotherapy, high CD38 expression on MM cells seems satisfactory to maximize the clinical efficacy such as the combination treatment of CD16F158V CD38^low^ NK cells and Daratumumab 120.

The natural CD38^low^ NK cells account for a small fraction of blood NK cells and their generation in high numbers is clinically challenging. However, the CRISPR/Cas9 technology to delete CD38 on NK cells (CD38KO NK) can be used to enhance the capacity of NK-mediated ADCC during CD38 mAb therapy. These CD38KO NK cells are resistant to fratricide, showing superior persistence in immune-deficient mice pretreated with CD38 antibodies and enhancing ADCC activity against CD38^+^ MM cell lines and primary MM cells. To maximize the CD38 mAb-mediated ADCC, the combination of CD38 mAb and CD38KO NK maintains NK cell function during Daratumumab therapy. This combined treatment has other benefits, including changes in aerobic metabolism with higher mitochondrial respiratory capacity of CD38KO NK cells. These findings have demonstrated that adoptive immunotherapy using *ex vivo* expanded CD38KO NK cells can boost Daratumumab activity in MM 90.

FT538 is the first multiplexed engineered NK cell therapy generated by CRISPR/Cas9. Its high-affinity CD16F158V and IL-15/IL-15 receptor fusion promote a high level of the immune response, and the CD38KO property makes it possible to mitigate NK cell fratricide by CD38 mAb. FT538 therapy combined with Daratumumab against MM cells demonstrates a more effective MM control than Daratumumab therapy alone 123. Recently a novel off-the-shelf product FT576 has come into our sight. FT576 comprises an anti-BCMA CAR, a high-affinity non-cleavable CD16, CD38 knockdown and IL-15/IL-15 receptor and has proved competent for ADCC against MM cells.

It shows great capacity of immune response of CD38-deficient g^-^NK cells in MM. In addition to Fate's work with the FT538 and FT576 products, Indapta Therapeutics has shown that CD38-deficient g-NK cells can be preferentially expanded which could produce high efficacy in combination with daratumumab as an anti-myeloma product without resorting to genetic engineering 94. The g-NK cell expresses infrequent NKG2A, upregulated NKG2C and elevated level of inhibitor of apoptosis proteins such as Bcl-2 53, 94. Compared with the performance of cNK cells in Daratumumab therapy, g-NK shows a greater CD38 mAb-induced cytotoxicity and improved persistence. The increased expression of perforin, granzyme, CD107a, IFN-γ and TNF-α in g-NK cells with the presence of Daratumumab are responsible for such phenomena 53. The great level of ADCC induced by Daratumumab with accumulation of activating signals could overcome the inhibitory effects even when the NK cells are not HLA-mismatched. Besides, the cytolytic enzymes in g-NK cells are activated, inducing MM cell apoptosis after CD16 crosslinking. Furthermore, a great increase in the number of g-NK cells is observed in the mice receiving Daratumumab treatment, which is 10 times higher than that of cNK cells in peripheral blood 94.

G^-^NK cells also prevent immune evasion of MM cells. Some MM cell lines escape from immune system by upregulation of surficial HLA-E. HLA-E could inhibit the antitumor activity of IL2-activated NK cells by binding with NKG2A. Thus, the lack of NKG2A on g^-^NK cells could cut off the HLA-E evasion 124, 125. G^-^NK cells share similar benefits in aerobic metabolism reprograming with CD38KO cells as well. Meanwhile, it shows great capacity of immune response in MM compared to KHYG1 cells for its potential against HLA-E mediated immune evasion. Since its potential efficacy and stable persistence, g^-^NK cells could be an excellent off-the-shelf product against R/R MM (Figure 6d) 94.

## Discussion

CD38 mAb, with its impressive single-agent activity to kill MM cells, has attracted increasing attention. However, the outcome of MM patients with Daratumumab is uncertain, partly due to its confounding effects on NK cell lines, such as the clearance of CD38^high^ NK cells and ADCC mainly mediated by NK cells with low or no CD38 membrane expression. The heterogeneity of NK cells with respect to quantity and functional changes might be related to the different outcomes in MM patients. Therefore, therapies combined with other treatments are considered helpful to maximize CD38 mAb effects against MM.

The combination of the CD38 mAb with an IMiD remarkably enhances NK cell-mediated ADCC by increasing the quantity and activity of NK cells, promoting the tumoricidal activity of macrophages, and enhancing CD38 mAb-mediated ADCP. Besides, another therapy combined with PIs improves overall response and progression-free survival among relapsed and refractory MM patients. But we have to pay attention to its infusion-related reactions and accounts for higher risk of thrombocytopenia and neutropenia. Combining the two antibodies, anti CD38 and anti PD-L1/PD-1, is beneficial for MM patients resistant to monotherapy. Furthermore, the cellular therapy with KHYG1, CD38KO NK cells or g^-^NK cells protects the immune activity of NK cells by upregulating the quantity or activity of CD38^low/-^ NK cells and preventing the HLA-E-mediated immune evasion to maximize the effects of CD38 mAb against MM.

CD38 mAb is a milestone in MM treatment and the future of combined therapy will be more promising if more in-depth studies are conducted. For instance, there is no effective method to select patients who will have a successful outcome in the long term after CD38 mAb therapy and exclude those who will not benefit from the treatment. The management guidance on precision medicine for different patients is still lacking. Guidelines for various treatments for different groups of patients with relapsed or refractory MM and drug-resistant MM, including single-agent and combined therapies, are needed to set clinical standards and reduce the economic burden on patients.

## Figures and Tables

**Table 1:** Selected ongoing and planned clinical trials on CD38 MoAb monotherapies and combined therapies in multiple myeloma

Study	Phase	Patient	Treatment
NCT04151667	2	NDMM in older adults (>65 years old)	IberDd vs DRd vs DVd
NCT03901963	3	NDMM with minimal residual disease positive after frontline ASCT	Daratumumab and Lenalidomide vs Lenalidomide alone
NCT02316106	2	SMM	Single agent 3 Daratumumab dose schedules (long, intermediate and short treatment duration)
NCT04975997	3	RRMM	IberDd vs DVd
NCT02807454	2	RRMM	Daratumumab and Durvalumab (PD-L1 MoAb) vs Pomalidomide, Daratumumab, Durvalumab and Dexamethasone
NCT02076009	3	RRMM	DRd vs Lenalidomide and Dexamethasone
NCT04614636	1	RRMM	FT538 monotherapy vs FT538 and Daratumumab vs FT538 and Elotuzumab (SLAMF7 MoAb)
NCT04700176	2	MM Previously exposed to Daratumumab-Based Regimens	Progressed on DRd to be treated with DPd and ATRA vs progressed on DPd to be treated with DPd and ATRA

IberDd, Daratumumab and Dexamethasone; DVd, Daratumumab, Bortezomib, and Dexamethasone; DRd, Daratumumab, Lenalidomide and Dexamethasone; DPd, Daratumumab, Pomalidomide, and Dexamethasone; RRMM, relapsed or refractory multiple myeloma; ATRA, all-trans retinoic acid; NDMM, newly diagnosed multiple myeloma; SMM, smoldering multiple myeloma; ASCT, autologous stem cell transplant.

## Abbreviations

MM: multiple myeloma
mAbs: monoclonal antibodies
IMiDs: immunomodulatory imide drugs
PIs: proteasome inhibitors
PD-1: programmed death 1
PD-L1: programmed death ligand 1
R/R MM: relapsed or refractory multiple myeloma
Tregs: regulatory T cells
NK: natural killer
FDA: Food and Drug Administration
ADCC: antibody-dependent cellular cytotoxicity
PBMCs: peripheral blood mononuclear cells
Bregs: regulatory B cells
MDSCs: myeloid-derived suppressor cells
NAD: nicotinamide adenine dinucleotide
ADPRC: adenosine diphosphate ribose cyclase
cADPR: cyclic adenosine diphosphate ribose
ADPR: adenosine diphosphate ribose
NAADP: Nicotinic acid adenine dinucleotide phosphate
NADP: nicotinamide adenine dinucleotide phosphate
TRPM2: transient receptor potential melastatin-2
ADP: adenosine diphosphate
ADO: adenosine
TCA: tricarboxylic acid
TNFR: tumor necrosis factor receptor
TRAIL: TNF-related apoptosis-inducing ligand
TRAIL-R: TNF-related apoptosis-inducing ligand-receptor
FasL: Fas ligand
FADD: Fas-associated death domain
NCRs: natural cytotoxicity receptors
FcγR: Fcγ receptor
HLA-I: human leukocyte antigen class I
ITAMs: immunoreceptor tyrosine-based activation motifs
MGUS: monoclonal gammopathy of undetermined significance
DNAM-1: DNAX accessory molecule-1
cNK: conventional NK
ZAP70: zeta-chain-associated protein kinase 70
MAPK: mitogen-activated protein kinase
IL-2: interleukin-2
CMV: Cytomegalovirus
CDC: complement dependent cytotoxicity
MAC: membrane attack complexes
BMSCs: bone marrow-derived mesenchymal stem/stromal cells
IFN-γ: interferon-γ
GM-CSF: granulocyte-macrophage colony-stimulating factor
ADCP: antibody-dependent cellular phagocytosis
PCD: programmed cell death
ROS: reactive oxygen species
pDC: plasmacytoid dendritic cells
ASCT: autologous stem cell transplantation
JAK1: janus kinase 1
STAT1/2: signal transducers and activators of transcription 1/2
IRF9: interferon regulatory factor 9
IFNs: interferons
ISGF3: IFN-stimulated gene factor 3
ISGs: interferon-stimulated genes
DR5: death receptor 5
SHP-2: Src homology region 2 domain-containing protein tyrosine phosphatase
PKC: protein kinase C
PI3K: phosphoinositide 3-kinase
ERK: extracellular-signal-regulated kinase
AKT: protein kinase B
ATRA: all-trans-Retinoic acid
ADRP: ADP-ribose pyrophosphate
KIR: Killer-cell immunoglobulin-like receptors
